# Apparent diffusion coefficient histogram in the differentiation of benign and malignant testicular tumors

**DOI:** 10.7150/ijms.88486

**Published:** 2024-01-01

**Authors:** Nguyen Dinh Minh, Nguyen Duy Hung, Trinh Anh Tuan, Ma Mai Hien, Ho Xuan Tuan, Nguyen Minh Duc

**Affiliations:** 1 Department of Radiology, Viet Duc Hospital, Hanoi, Vietnam.; 2 Department of Radiology, Hanoi Medical University, Hanoi, Vietnam.; 3 Department of Medical Imaging, Da Nang University of Medical Technology and Pharmacy, Vietnam.; 4 Department of Radiology, Pham Ngoc Thach University of Medicine, Ho Chi Minh City, Vietnam.

**Keywords:** testicular cancer, benign testicular tumor, malignant testicular tumor, diffusion-weighted imaging, apparent diffusion coefficient maps, histogram analysis

## Abstract

**Purpose:** This retrospective study assessed the value of histogram parameters of the apparent diffusion coefficient (ADC) map (HA) in differentiating between benign and malignant testicular tumors. We compared the diagnostic performance of two different volume-of-interest (VOI) placement methods: VOI 1, the entire tumor; VOI 2, the tumor excluding its cystic, calcified, hemorrhagic, and necrotic portions.

**Materials and methods:** We retrospectively evaluated 45 patients with testicular tumors examined with scrotal contrast-enhanced magnetic resonance imaging. These patients underwent surgery with the pathological result of seven benign and 39 malignant tumors. We calculated the HA parameters, including mean, median, maximum, minimum, kurtosis, skewness, entropy, standard deviation (SD), mean of positive pixels, and uniformity of positive pixels by the two different VOI segmentation methods. We compared these parameters using the chi-square test, Mann-Whitney U test, and area under the receiver operating characteristic curve (AUC) to determine their optimal cut-off, sensitivity (Se), and specificity (Sp).

**Result:** This study included 45 patients with 46 testicular lesions (seven benign and 39 malignant tumors), one of which had bilateral testicular seminoma. With the VOI 1 method, benign lesions had significantly lower maximum ADC (*p* = 0.002), ADC skewness (*p* = 0.017), and ADC variance (*p* = 0.000) than malignant lesions. In contrast, their minimum ADC was significantly higher ADC (*p* = 0.000). With the VOI 2 method, the benign lesions had significantly higher ADC SD (*p* = 0.048) and maximum ADC (*p* = 0.015) than malignant lesions. In contrast, their minimum ADC was significantly lower (*p* = 0.000). With the VOI 1 method, maximum ADC, ADC variance, and ADC skewness performed well in differentiating benign and malignant testicular lesions with cut-offs (Se, Sp, AUC) of 1846.000 (74.4%, 100%, 0.883), 39198.387 (79.5%, 85.7%, 0.868), and 0.893 (48.7%, 100%, 0.758).

**Conclusion:** The HA parameters showed value in differentiating benign and malignant testicular neoplasms. The entire tumor VOI placement method was preferable to the VOI placement method excluding cystic, calcified, hemorrhagic, and necrotic portions in measuring HA parameters. Using this VOI segmentation, maximum ADC performed best in discriminating benign and malignant testicular lesions, followed by ADC variance and skewness.

## Introduction

Testicular tumor is a rare entity representing approximately 1%-1.5% of tumors in men, but it is the most common tumor in patients aged 15-44 years 1. While 95% of testicular tumors are malignant, the incidence of benign tumors tends to increase with the usefulness of imaging diagnosis 1,2. For malignant lesions, total testicular resection with or without chemoradiotherapy, depending on histopathologic results, is considered the optimal treatment 3. In contrast, for benign lesions, tumor resection with preservation of normal testicular parenchyma helps to maintain endocrine and reproductive functions 1. Therefore, discrimination between benign and malignant neoplasms plays a pivotal role in treatment management and in avoiding unnecessary overtreatment in benign cases.

While biopsy is considered the gold standard in differentiating benign and malignant tumors, it is an invasive procedure with potential hemorrhage or infection risks and limitations in evaluating the extent of lesions 2. Ultrasound with a high-frequency linear probe (7-10 MHz) is the initial imaging diagnostic tool in screening testicular pathology, with undeniable advantages such as determining intra- or extra-testicular masses and discriminating tumors from non-tumor lesions such as infections or testicular torsions. However, the value of conventional ultrasound, contrast-enhanced ultrasound, and elastography in differentiating benign and malignant tumors and evaluating the extent of lesions remains controversial 4 5,6.

Scrotal magnetic resonance imaging (MRI) is an extremely valuable imaging modality for assessing testicular pathologies, including testicular neoplasms, as recommended by the European Society of Urogenital Radiology 7. This non-invasive tool with multiple sequences and planes, combined with the injection of a contrast agent, plays a vital role in determining tumor components such as fat, calcification, necrosis, hemorrhage, and local extent, thereby aiming at determining the lesion's nature 8.

Diffusion-weighted imaging (DWI) with the quantification of parameters for the apparent diffusion coefficient (ADC) map through region-of-interest (ROI) segmentation is increasingly used to differentiate benign and malignant tumors in many organs, including the testicles. According to Wang et al., ADC value ≤ 0.90 × 10-3 mm^2^/s suggests a malignant tumor 2. However, ADC-map-based measurements vary according to ROI location and size 9. Histogram analysis of ADC maps (HA) is a recent method applied in distinguishing two groups based on statistical indicators using volume-of-interest (VOI) placement to cover the entire tumor. This method has advantages over placing the ROI on a single slice or localized site in determining tumor heterogeneity, including hemorrhage and necrosis (a common finding in malignant tumors), helping to better distinguish between two groups 1. Therefore, this study evaluated the diagnostic value of HA parameters in discriminating benign and malignant neoplasms using two VOI placement methods, one using the entire tumor and the other excluding its cystic, necrotic, calcified, and hemorrhagic portions.

## Methods

### Study population

This retrospective study was conducted in the Viet Duc University Hospital Radiology Department between January 2019 and June 2023 and included 45 patients with testicular neoplasms. All research subjects were examined with contrast-enhanced scrotal MRI and then underwent surgery with a pathological result of benign or malignant tumors. The exclusion criteria included patients with a previous history of biopsy or treatment (radiotherapy or operative tumor resection) and any artifacts that affect the quality of MRI images and prevent accurate measurements. This study was performed according to the Declaration of Helsinki. All procedures were conducted according to appropriate laws and regulations. The patient or their legal representative provided informed consent to participate in this study.

### Technique

The patients underwent contrast-enhanced scrotal MRI using a 3.0 Tesla (SIGNA Pioneer MR; GE Healthcare, USA) or 1.5 Tesla (Gyroscan and Intera, Philips Healthcare) scan system. The same technique and protocol were used for all patients (Table 1).

DWI was performed on the axial plane with a repetition time (TR) of 4000 ms, echo time (TE) of 60 ms, Flip angle of 90^o^, slice thickness of 8 mm, field of view (FOV) of 340, matrix of 128×128, and two *b* values (0 and 1000) 11

The ADC map assesses the diffusion capacity of intracellular water molecules according to the formula S(b)/S(50) = exp[-(b - 50) ×ADC], where S(b) is the signal intensity at a *b*-value of 1000 s/mm^2^, and S(50) is the signal intensity at a *b*-value of 50 s/mm^2^.

### Image analysis

MRI images were assessed retrospectively, transferred from the Picture Archiving and Communication System workstation to a personal computer, and converted into the DCM format. The VOI measurement was performed with The Medical Imaging Interaction Toolkit software (MITK Workbench v2022.10; Division of Medical Image Computing, German Cancer Research Center, Heidelberg, Germany) by two urogenital radiologists with at least six years of experience and no knowledge of the final pathologic results; disagreements were resolved by discussion.

The boundaries of entire tumors were evaluated using the ADC maps at a *b*-value of 1000 s/mm^2^.

We determined the following regions in conventional MRI images 2,7,12:

1. Tumoral hemorrhagic or calcified portions: hyperintense on T1W.

2. Tumoral cystic degeneration or necrotic portions: significantly hypointense on T1W, hyperintense on T2W, and unenhanced in contrast-enhanced T1W.

3. The normal testicular parenchyma: the contralateral normal testicular parenchyma in the same slide.

We measured HA using two different VOI placement methods:

1. VOI 1: the VOI was manually drawn on each slide of the entire tumor boundary, containing cystic, hemorrhagic, calcified, and necrotic portions.

2. VOI 2: the VOI was manually drawn on each section of the entire tumor, excluding cystic, hemorrhagic, calcified, and necrotic portions.

Then, we calculated the following accumulated ADC parameters: mean, maximum (max), minimum (min), kurtosis, skewness, entropy, standard deviation (SD), mean of positive pixels (MPP), and uniformity of positive pixels (UPP).

### Histopathological examination

All histopathological results were classified as benign and malignant tumors according to The World Health Organization 2022 criteria 13.

### Statistical analysis

The data were analyzed using SPSS (version 20.0; Chicago, IL, USA) to assess the correlation between HA parameters and the pathological features of testicular neoplasms. Parameters with a normal distribution (*p* > 0.005) are presented as mean ± SD and compared using the Chi-square or Fisher's exact test. Parameters with a nonnormal distribution (*p* < 0.005) are presented as the median (25^th^-75^th^ percentiles) and compared using the Mann-Whitney U test. A *p*-value of <0.005 was considered statistically significant.

A receiver operating characteristic (ROC) curve was created to determine the optimal cut-off for each parameter by maximizing the sum of sensitivity (Sn) and specificity (Sp) using the Youden index.

## Results

The retrospective study included 45 patients with 46 testicular tumors (seven benign and 39 malignant), of which one 61-year-old patient had bilateral testicular seminoma. The mean age was 24.57 ± 11.0 years in the benign group and 36.10 ± 11.72 years in the malignant group; the youngest patients were 7 and 20 years old, respectively, and the oldest patients were 43 and 65 years old, respectively. The age distribution did not differ significantly between the two groups (*p* = 0.409).

The distribution of benign and malignant testicular tumors is shown in Table 2. The benign tumor group included seven cases, of which four had epidermoid cysts, two had Leydig cells neoplasms, and one had a dermoid cyst. The malignant tumor group included 39 cases, of which 19 had seminoma, 16 had non-seminoma germ cell tumors, and four had lymphomas. The pathological descriptions are summarized in Table 2.

**Figure 3:**
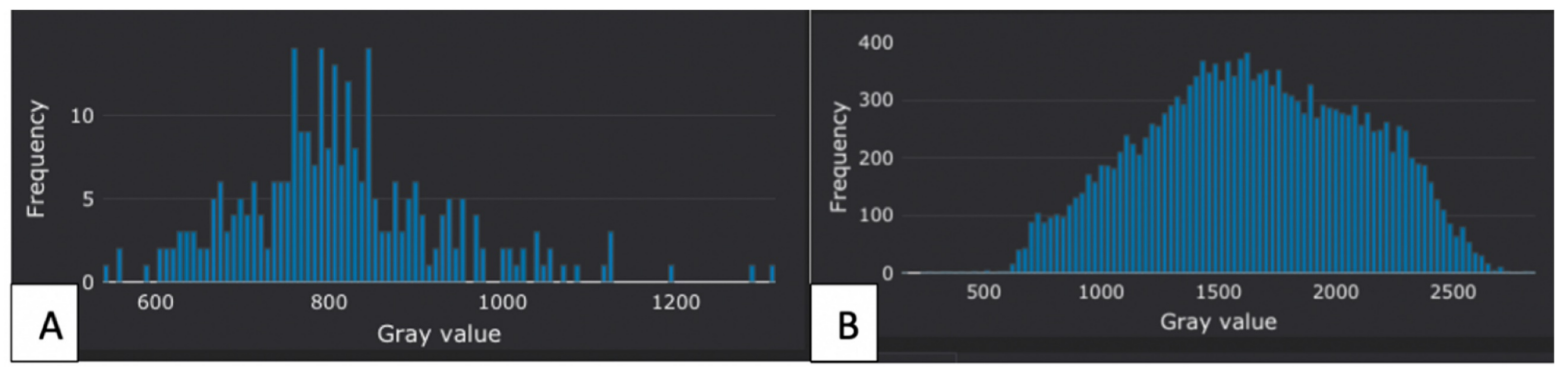
Representative histograms for one case in the benign and malignant tumor groups. (A) A histogram of a 25-year-old man with the histopathological result of an epidermoid cyst. (B) A histogram of a 44-year-old man with seminoma.

Figure 3 shows a representative histogram of one case in the benign and malignant tumor groups after being processed by the software.

Table 3 compares HA parameters using the two VOI placements. With the VOI 1 method, ADC max (*p* = 0.002), skewness (*p* = 0.017), and variance (*p* = 0.000) were significantly lower in the benign group than in the malignant group, while ADC min (*p* = 0.000) was significantly higher. With the VOI 2 method, ADC SD (*p* = 0.048) and max (*p* = 0.015) were significantly lower in the benign group than in the malignant group, while ADC min (*p* = 0.000) was significantly higher.

**Figure 4:**
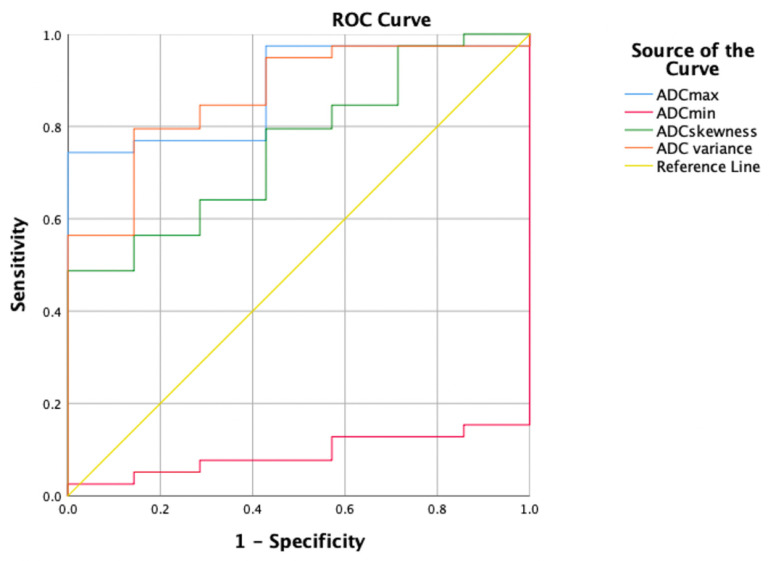
The ROC curves for the HA parameters with the VOI 1 placement method.

The ROC curves for the HA parameters with the VOI 1 placement method are shown in Table 4 and Figure 4. ADC max had the highest diagnostic ability (area under the ROC curve [AUC] = 0.883), followed by ADC variance (AUC = 0.868), ADC skewness (AUC = 0.758), and ADC min (AUC = 0.092). The optimal cut-off for ADC max was 1846.000, with a Sn of 74.4%, Sp of 100%, and Youden index of 0.744.

**Figure 5:**
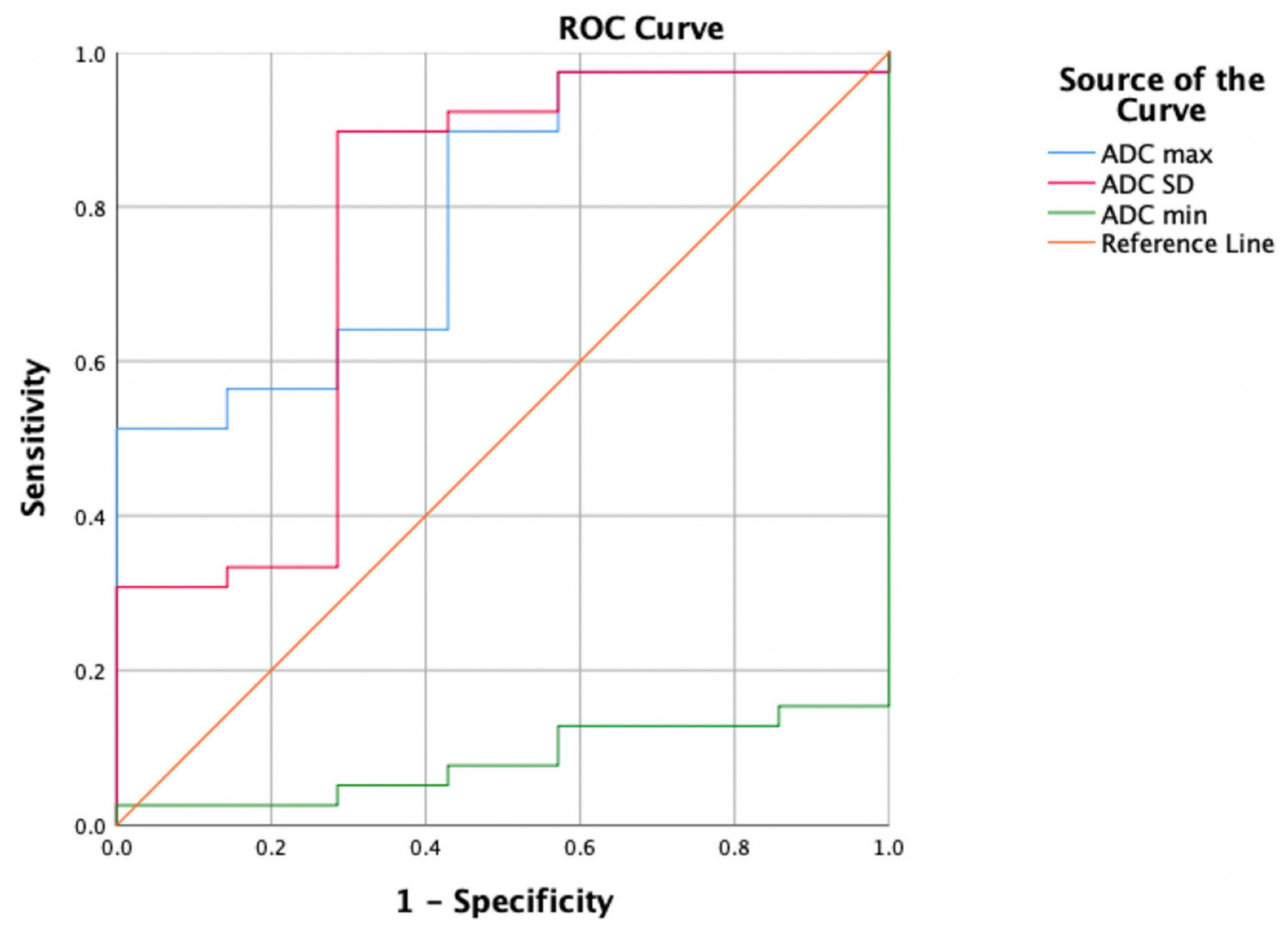
The ROC curves for the HA parameters with the VOI 2 placement method.

The ROC curves for the HA parameters with the VOI 2 placement method are shown in Table 5 and Figure 5. ADC max had the highest diagnostic ability (AUC = 0.791), followed by ADC SD (AUC = 0.769) and ADC min (AUC = 0.084). The optimal cut-off for ADC max was 1693.500, with a Sn of 51.3%, Sp of 100%, and Youden index of 0.513.

## Discussion

HA analysis is a quantitative method for evaluating the statistical parameters in each image based on VOI placement to cover the entire tumor. Therefore, the histogram's shape and asymmetry reflect microstructural differences in tumor composition corresponding to histopathological grade, distinguishing benign and malignant tumors 8,9. Previous studies have shown that placing the VOI so that it covers the entire tumor volume allows for a better assessment than the previous approach of placing an ROI on a specific slice or localized tumor region, often avoiding its cystic, necrotic, and hemorrhagic portions, leading to limitations in reflecting its heterogeneity, especially for malignant tumors that have more necrotic and bleeding portions than benign tumors 8,14. This study examined 45 patients with 46 testicular tumors (seven benign and 39 malignant) to evaluate the diagnostic value of HA in discriminating benign and malignant neoplasms with two VOI placement methods, one using the entire tumor and the other excluding its cystic, necrotic, calcified, and hemorrhagic portions.

We compared each parameter's AUC value with the two VOI placement methods, finding that those for ADC max and variance using VOI 1 (0.883 and 0.868, respectively) tended to be significantly larger than those of ADC max and SD using VOI 2 (0.791 and 0.769, respectively). Apart from these parameters, ADC skewness also showed a high AUC of 0.758 with VOI 1. Therefore, our results suggest that the VOI 1 placement method likely improved the ability to differentiate between benign and malignant tumors. Our results are similar to those of Pederson et al., which studied a group of malignant testicular tumors consisting of 26 tumors and pointed out the highest value of whole-tumor VOI placement in diagnosing testicular tumors 9.

Our study's results differ from Fan et al., who used the VOI 1 placement method in 61 testicular lesions. They found that ADC max was not a reliable indicator for distinguishing benign and malignant lesions 1. In the group of 18 benign lesions in Fan's study, five out of 18 cases (27.78%) were non-tumors (including four cases of inflammation and one case of testicular torsion), which could explain the different ADC max findings between studies 1.

We evaluated HA parameters using the VOI 1 method. ADC max (*p* = 0.002), ADC (*p* = 0.017), and variance (*p* = 0.000) were significantly lower in the benign group than in the malignant group, while ADC min was significantly higher (*p* = 0.000). Skewness indicates asymmetry, and variance indicates dispersion. There was greater homogeneity in the benign group than in the malignant group, so its histogram was sharper. Therefore, the skewness and variance in the homogeneous group will be lower than in the less homogeneous group. Our results are similar to those of Min et al., who compared a more homogeneous group of seminomas with a less homogeneous group of non-seminomas 15.

In our study, with both VOI placement methods, ADC min was consistently greater in the benign tumor group than in the malignant tumor group. Previous studies also showed that ADC min was significantly lower in the malignant testicular lesion group than in the benign group 1. However, we found that ADC min was unreliable in discriminating the two groups since its AUC was 0.092 with VOI 1 and 0.084 with VOI 2. This finding differs from Fan et al. 1. Our study's benign group included four cases with epidermoid cysts (57.14%), a benign tumor with diffusion restriction and a low ADC. Consequently, the ADC min value in our benign group might be closer to our malignant group 16, potentially explaining the difference between our findings and those of Fan et al.

Among the HA parameters, ADC max had the highest diagnostic value in differentially diagnosing benign and malignant tumors with an AUC (cut-off, Sn, Sp) of 0.883 (1846.000, 74.4%, 100%), followed by ADC variance. Our study is the first to note the valuable role of ADC max in distinguishing between benign and malignant tumors. In addition, we encountered one patient with bilateral testicular seminoma in our study. Testicular neoplasm is rare, with tumors in both testicles even rarer, accounting for about 1%-2% of testicular masses diagnosed. Tumors in both testicles in the same patient often share similar histology and pathology; most are seminoma 17.

Our study had certain limitations. Firstly, its sample size was relatively small, which may affect the representativeness of its results. Secondly, its research subjects underwent scrotal MRI with 3.0 Tesla or 1.5 Tesla scan systems. Therefore, future studies should include a larger number of patients examined using the same imaging system. In this study, the VOI was manually drawn on each slide of the entire tumor, so there is a risk of error due to drawing VOI on the benign part and affecting the results, this is the reason why we placed VOI carefully, repeated the calculation at least 3 times to minimize the errors.

## Conclusions

The HA parameters, essentially ADC max showed diagnostic value in differentiating benign and malignant testicular masses. The entire tumor VOI placement method was preferable to the VOI placement method excluding cystic, calcified, hemorrhagic, and necrotic portions in measuring HA parameters. Using this VOI placement method, ADC max performed best in discriminating benign and malignant testicular lesions, followed by ADC variance and skewness.

## Figures and Tables

**Figure 1:**
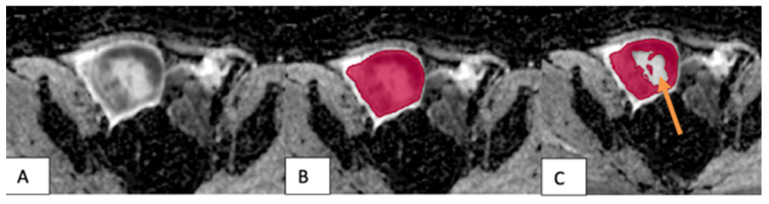
The VOI placement method in malignant tumors. (A) A tumor in the right testicle showed uneven hypointensity on the ADC map. (B) The VOI 1 method (red) covered the entire tumor, including its cystic and necrotic portions. (C) The VOI 2 method excluded the tumor's cystic and necrotic portions (arrow).

**Figure 2:**
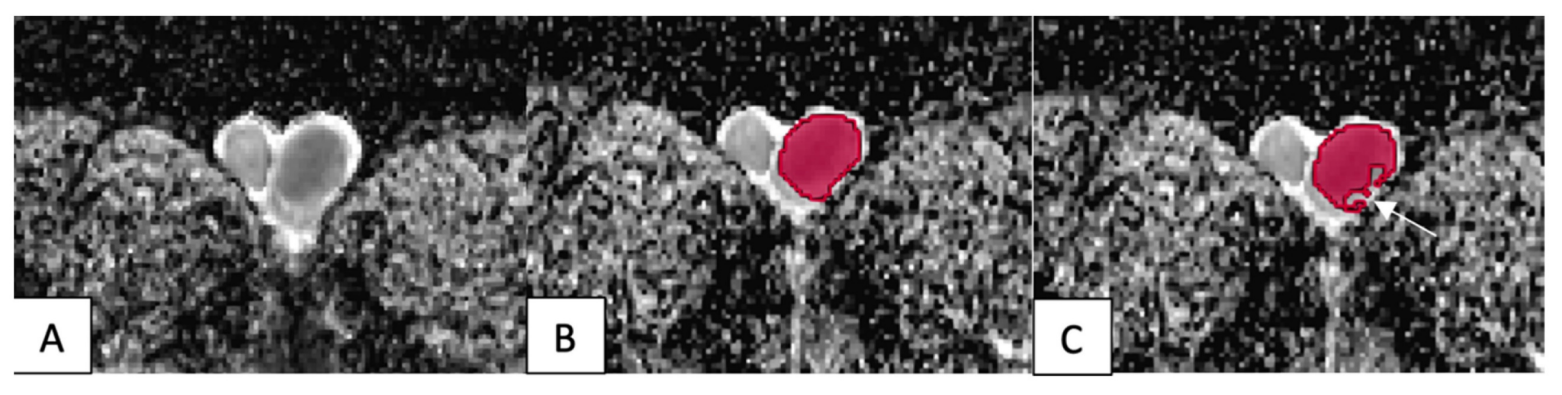
The VOI placement method in benign tumors: (A) A tumor in the left testicle showed slight hypointensity on the ADC map. (B) The VOI 1 method (red) covered the entire tumor, including its cystic and necrotic portions. (C) The VOI 2 method excluded the tumor's cystic and necrotic portions (arrow).

**Table 1:** Imaging protocol for scrotal MRI using 3.0 Tesla 4 and 1.5 Tesla 10.

Sequence	Plane	(Tesla)	TR (ms)	TE (ms)	Gap (mm)	Thickness (mm)	FOV	Matrix
T1W	Axial	1.5	650	15	0.5	3	240x270	180x256
		3.0	500-700	8-10	3	3	180x180	200-250 x 300-350
T2 TSE	Axial Coronal Sagittal	1.5	4000	100	0.5	3	240x270	180x256
		3.0	5000-7000	100-120	3.3	3	200x200	300-340x200-250
DWI	Axial	1.5	4000	60	8	2	340x340	128x128
		3.0	3000	100	0.5	3	220x176	90x90
T1W CE+	Axial	1.5	650	15	0.5	3	240x270	180x256
		3.0	500-700	8-10	3.3	3	180x180	240-260x200-220

FOV: field of view; T2 TSE: T2-weighted turbo spin-echo; T1W: T1-weighted spin echo; CE+: a single dose of intravenous contrast agent injection (Gadolinium-DTPA [0.2 mmol/kg] at an injection rate of 2.0 mL/s).

**Table 2:** Descriptive statistics for pathological descriptions.

Benign neoplasm	Number of lesions	Volume mm^3^	Malignant neoplasm	Number of lesions	Volume mm^3^
Epidermoid Cyst	4	8229.62	Seminoma	19	96780.09
Dermoid Cyst	1	1240.93	Mixed germ cell tumor	12	78834.75
Leydig cells tumor	2	882.78	Yolk sac tumor	2	40545.76
			Teratoma	2	37222.85
			Lymphoma	4	171898.06
Total	7		Total	39	

**Table 3:** Comparison of HA parameters between the two VOI placement methods.

Parameter	Method	Benign neoplasm	Malignant neoplasm	P value
**ADC mean**	VOI 1	917.78±185.03	1006.87±302.47	0.457
	VOI 2	878.37±170.79	849.96±230.85	0.758
**ADC median**	VOI 1	916.70±190.53	947.80±325.51	0.808
	VOI 2	877.94±183.92	825.13±242.88	0.588
**ADC SD**	VOI 1	150.23±60.99	322.01±157.59	0.007
	VOI 2	146.04±74.68	206.10±71.49	0.048*
**ADC MPP**	VOI 1	917.78±185.03	1008.31±303.46	0.451
	VOI 2	878.37±170.79	850.93±230.67	0.766
**ADC max**	VOI 1	1366.57±355.82	2318.36±751.62	0.002*
	VOI 2	1282.86±325.87	1824.49±545.14	0.015*
**ADC min**	VOI 1	583.86±64.49	249.59±200.80	0.000*
	VOI 2	598.14±72.52	280.82±200.18	0.000*
**ADC skewness**	VOI 1	0.22±0.47	0.84±0.63	0.017*
	VOI 2	0.39±0.38	0.83±0.86	0.195
**ADC kurtosis**	VOI 1	3.37±0.75	4.29±1.96	0.231
	VOI 2	3.17±0.98	5.15±2.84	0.077
**ADC UPP**	VOI 1	0.05±0.04	0.03±0.01	0.252
	VOI 2	0.05±0.04	0.03±0.01	0.191
**ADC Entropy**	VOI 1	4.97±1.09	5.59±0.43	0.189
	VOI 2	4.70±1.06	5.39±0.50	0.139
**ADC Uniformity**	VOI 1	0.05±0.04	0.03±0.01	0.252
	VOI 2	0.05±0.04	0.03±0.01	0.191
**ADC Variance**	VOI 1	25757.91±20992.12	127890.64±126698.45	0.000*
	VOI 2	26109.01±25506.31	47454.96±32173.35	0.104

Parameters were tested for normality using the Kolmogorov-Smirnov test with a *p*-value of >0.05 indicating they followed a normal distribution. Normally distributed parameters are presented as mean ± SD and compared using the Chi-square test. *: *p*-value < 0.05.

**Table 4:** The ROC curves for the HA parameters using the VOI 1 placement method.

Parameter	AUC	Cut-off	Sn	Sp	Youden Index
**ADC max**	0.883	1846.000	74,4	100	0,744
**ADC variance**	0.868	39198.387	79,5	85,7	0,652
**ADC skewness**	0.758	0.893	48,7	100	0,487

**Table 5:** The ROC curves for the HA parameters with the VOI 2 placement method.

Parameter	AUC	Cut-off	Sn	Sp	Youden Index
**ADC max**	0.791	1693.500	51.3	100	0.513
**ADC SD**	0.769	130.376	89.7	71.4	0.611
